# Comparative Analysis of Genome-Wide Chromosomal Histone Modification Patterns in Maize Cultivars and Their Wild Relatives

**DOI:** 10.1371/journal.pone.0097364

**Published:** 2014-05-12

**Authors:** Shibin He, Shihan Yan, Pu Wang, Wei Zhu, Xiangwu Wang, Yao Shen, Kejia Shao, Haiping Xin, Shaohua Li, Lijia Li

**Affiliations:** 1 State Key Laboratory of Hybrid Rice, College of Life Sciences, Wuhan University, Wuhan, China; 2 State Key Laboratory of Cotton Biology, College of Life Sciences, Henan University, Kaifeng, China; 3 Key Laboratory of Plant Germplasm Enhancement and Specialty Agriculture, Wuhan Botanical Garden, The Chinese Academy of Sciences, Wuhan, China; 4 School of Physics and Electronics, Henan University, Kaifeng, China; Oregon State University, United States of America

## Abstract

Recent advances demonstrate that epigenome changes can also cause phenotypic diversity and can be heritable across generations, indicating that they may play an important role in evolutionary processes. In this study, we analyzed the chromosomal distribution of several histone modifications in five elite maize cultivars (B73, Mo17, Chang7-2, Zheng58, ZD958) and their two wild relatives (*Zea mays* L. ssp. *parviglumis* and *Zea nicaraguensis*) using a three-dimensional (3D) epigenome karyotyping approach by combining immunostaining and 3D reconstruction with deconvolution techniques. The distribution of these histone modifications along chromosomes demonstrated that the histone modification patterns are conserved at the chromosomal level and have not changed significantly following domestication. The comparison of histone modification patterns between metaphase chromosomes and interphase nuclei showed that some of the histone modifications were retained as the cell progressed from interphase into metaphase, although remodelling existed. This study will increase comprehension of the function of epigenetic modifications in the structure and evolution of the maize genome.

## Introduction

Chromatin in eukaryotes is composed of DNA and its associated core histones and is subject to various post-translational modifications in the amino-terminal tails of histones and methylation in the cytosine residues of DNA [1]. Some modifications such as histone acetylation and H3K4 methylation are enriched in euchromatin, while H3K9 methylation, H3K27 methylation, and DNA methylation are generally thought to be the marks of condensed heterochromatin [2], [3]. These epigenetic modifications have an effect on gene expression and phenotype and can be heritable across generations, indicating that they also play a role in evolution [4]–[6]. Although histones and their modifications are conserved, extensive studies have revealed that there are some differences in the distributions and functional meanings of histone modifications between the genomes of animals and plants as well as between different plant species [2]. In mouse nuclei, H3K9 trimethylation and H4K20 trimethylation preferentially mark constitutive heterochromatin [7], whereas in *Arabidopsis thaliana*, they are not typical marks for heterochromatin [2]. H3K9me1, a heterochromatin-specific mark in angiosperms has been found to be enriched in euchromatic domains in gymnosperm species [8]. However, very little is known about how the histone modification patterns change during plant evolution from wild species to cultivated species.

Genome-wide analysis of the epigenome can be revealed at the molecular level by the chromatin immunoprecipitation-sequencing (ChIP-seq) technique [9]. However, highly repetitive DNAs hinder sequencing-based analysis of the plant genome and ChIP-seq is not suitable for non-sequenced genomes [10]. There are some changes in epigenetic states from interphase to metaphase, but ChIP-seq cannot distinguish cells in different phases of the cell cycle. Immunocytological analysis with antibodies specific to histone modification and DNA methylation is a powerful technique for identifying individual chromosomes and analysis of the entire epigenome at the chromosomal level [3]. The distribution of histone modifications can be traced along metaphase chromosomes of animals and plants by this technique [2], [3]. Many plants such as *A. thaliana* [11], *Zea mays* [12], *Vicia faba* [13] and *Secale cereale* [14] have been investigated so far with regard to the chromosomal distribution of histone modifications. To date, few systematic comparative analyses of epigenome between wild species and cultivated species have been performed at the cellular level. Exploring the variations in epigenetic patterns during plant evolution is very important for biological research and agriculture.

Maize (*Zea mays* L.) is one of the most important crops and is considered a model plant for studying genome evolution [15], [16]. In this study, we developed a three-dimensional (3D) epigenome karyotype method by combining immunostaining, 3D reconstruction and deconvolution techniques to analyse the entire epigenome at the metaphase chromosomal level in five elite maize cultivars (four elite inbred lines: B73, Mo17, Chang7-2, Zheng58; a hybrid: ZD958 (Chang7-2×Zheng58) and their two wild relatives (*Z. mays* L. ssp. *parviglumis* and *Z. nicaraguensis*), using six antibodies against modified histones (euchromatin-specific marks: H3K4me2, H3K4me3, H3K9ac, H4K5ac; heterochromatin-specific marks: H3K9me2, H3K27me2). We compared the distribution of these histone modifications along chromosomes among maize cultivars and their wild relatives and demonstrated that the histone modification distribution patterns are conserved at the chromosomal level during maize evolution. Comparison of the histone distribution patterns between metaphase and interphase showed that some of the interphase histone modifications were maintained as the cell progressed towards metaphase, although some remodelling of histone modifications is known to occur during mitosis.

## Materials and Methods

### Plant materials and preparation of metaphase chromosomes and interphase nuclei

Seeds of four elite maize inbred lines (B73, Mo17, Zheng58, Chang7-2), a high-yielding commercial hybrid ZD958 (Zheng58×Chang7-2) and two teosinte taxa (*Zea mays* L. ssp. *parviglumis* and *Zea nicaraguensis*) were germinated on wet filter paper in petri dishes at 28°C. Root tips of 0.5 to 1 cm length were excised, pre-treated in water-supersaturated α-bromonaphthalene in the dark at room temperature for 4 h, fixed in freshly prepared 4% (w/v) paraformaldehyde for 40 min at 4°C and then digested with an enzyme mixture of 2% cellulase and 2% pectolyase dissolved in 1×PBS buffer (pH 7.3) for about 1 h at 37°C. The digested root tips were squashed in a drop of 1×PBS on a slide below a coverslip. After removal of the coverslip, the slide was stored at −20°C. Nucleus isolation was performed according to the procedure described by Li et al. [17]. The samples were chopped with a blade in nucleus isolation buffer (10 mM MgSO_4_, 50 mM KCl, 5 mM HEPES, 1 mg/mL dithiothreitol, and 0.2% Triton X-100, pH 7.2) on ice. The nuclei were filtered through a 33 µm nylon mesh, fixed in 4% (w/v) paraformaldehyde for 30 min at 4°C, centrifuged (200×g, 10 min, 4°C), resuspended in isolation buffer and spread on a slide.

### Immunostaining and three-dimensional (3D) image reconstruction and deconvolution

Immunostaining was performed as described by Hu et al. [18]. The prepared slides were incubated in 1×PBS containing 3% BSA (w/v) for 1 h at 37°C, washed with 1×PBS three times (for 5 min each) and incubated with the primary antibodies listed in Table 1. After 12 h of incubation at 4°C and washing in 1×PBS, primary antibody was detected using an FITC-labelled goat anti-rabbit secondary antibody (12-507, Millipore, Billerica, MA, USA) diluted to 1∶100 with 1×PBS containing 3% BSA (w/v). Control experiments were carried out with the secondary antibody alone and did not show any signal. The specific of these commercial antibodies has been confirmed by Dot Blot and Western Blot analysis and Immunocytochemistry experiment by the company and these antibodies have been widely used in epigenetic analysis in plants [2], [8], [10]. Immunostained slides were mounted in Vectashield (Vector Laboratories, Burlingame, CA, USA) containing 5 µg/mL 4′,6-diamidino-2-phenylindole (DAPI, Sigma-Aldrich, St. Louis, MO, USA). Fluorescent signals were captured separately with appropriate filters using an Olympus BX60 fluorescence microscope (Shinjuku-ku, Tokyo, Japan) equipped with a Cool Snap HQ_2_ CCD camera (Photometrics, Tucson, AZ, USA). In order to better reflect the conformational features of chromatin and to obtain clear fluorescence banding patterns, we measured the height of chromosomes or nuclei, set the step length, and collected a group of two-dimensional (2D) images by controlling the Z-axis stepper. Image haze was removed using the ‘No Neighbours deconvolution’ command in MetaMorph 7.7.3 software (Molecular Devices, Sunnyvale, CA, USA). Finally, images were recovered using the ‘3D reconstruction’ command in the MetaMorph software and were then merged. The position of good chromosome spreads was recorded to re-evaluate after FISH.

**Table 1:** List of primary antibodies used in the present study.

Antibody	Source/Cat. No.	Host	Dilution
H3K4me2	Upstate/07-030	Rabbit	1∶200
H3K4me3	Upstate/07-473	Rabbit	1∶200
H3K9me2	Upstate/07-441	Rabbit	1∶100
H3K27me2	Abcam/ab24684	Rabbit	1∶200
H3K9ac	Upstate/07-352	Rabbit	1∶100
H4K5ac	Upstate/07-327	Rabbit	1∶200

### Fluorescence in situ hybridization (FISH) and re-probing

After immunostaining, the slides were washed with 1×PBS, post-fixed in Carnoy's solution (ethanol: acetic acid  = 3: 1, v/v) and dehydrated with absolute ethanol. Four repeated DNA sequences were used for FISH karyotyping (TAG microsatellite sequence, CentC, knob 180-bp sequence, and 45S rDNA) and labelled by nick translation with biotin-11-dUTP or digoxin-11-dUTP (Roche, Mannheim, Germany). FISH was performed as described by He et al. [19]. For sequential FISH, digoxin-11-dUTP-labelled TAG microsatellite sequences and biotin-11-dUTP-labelled CentC were applied in the first FISH. Digoxin-11-dUTP- and biotin-11-dUTP-labelled 180-bp knob sequence and biotin-11-dUTP-labelled 45S rDNA were applied in the second FISH. After the first hybridization and detection, the slide was washed gently with 1×PBS and 2×SSC respectively, submerged in 70% formamide at 70°C for 1 min and 50% formamide at 42°C for 9 min, and washed again with 2×SSC. Finally, the slide was rinsed with absolute ethanol and air dried for the second FISH. Images were captured and processed as above. After multiple pseudo-colorings, FISH karyotypes were established.

### Analysis of Chip-seq and DNA methylation data

Chip-seq (H3K4me2, H3K9me2) and DNA methylation data of the maize inbred line B73 were obtained from NCBI SRA (accession ID: SRR029396/7, SRR029398/9 and SRR029404/5/6). The reference genome of B73 was downloaded at www.maizesequence.org (release version 5b.60). Mapping reads of the three libraries were aligned to the reference genome using Bowtie. To generate precise results, we accepted only unique mapped reads which had less than 1 mismatch with the genome. The mean depth of each 10 Mb window (excluding ambiguous ‘N’ bases) was calculated to represent the signal intensity.

## Results

### Construction of 3D epikaryotypes and identification of individual maize chromosomes

**Figure 1:**
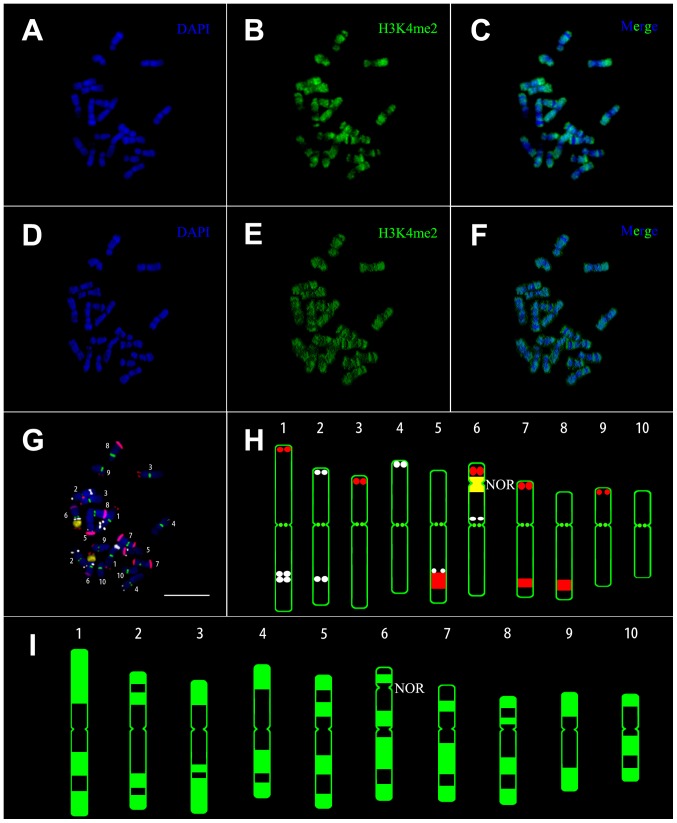
Karyotyping of immunolabelled metaphase chromosomes. (A-C) Original image of chromosomal distribution of H3K4me2 from a maize inbred line (chang7-2) (2D). (D-F) Enhancement of the contrast of the original image by three-dimensional reconstruction after deconvolution (3D). (A, D) 4, 6-diamidino-2-phenylindole (DAPI) staining signals in blue, (B, E) immunostaining signals in green and (C, F) merge of both. (G, H) FISH karyogram constructed from the same spread. Assignments of pseudo-colors to each probe: TAG as white, CentC as green, 45s rDNA as yellow and knob 180-bp as red. (G) Metaphase chromosome identification by combining the four probes and DAPI staining. (H) Ideogram of FISH karyotype indicating the position of the four probes. (I) Karyogram of the H3K4me2 profile constructed from the immunolabelled chromosomes by three-dimensional reconstruction after deconvolution. NOR: nucleolus organizing regions. Scale bar = 10 µm.

Conventional banding karyotypes generally reflect regional variations in DNA sequences and organization when DNA is stained with certain dyes [20]. Also, histones can be immunostained for karyotyping. However, in plants, immunostaining signals often do not appear as clear bands. Thus, we adapted a 3D-epigenome karyotyping method by combining immunostaining and 3D reconstruction with deconvolution techniques, which could improve banding resolution. In the original image (2D image) of an immunostained metaphase chromosome spread from a maize inbred line Chang7-2, DAPI staining signals were distributed along the whole chromosomes except at centromeres and secondary constrictions (Figure 1A). H3K4me2 signals were mainly enriched at the chromosome ends with consistent fluorescence intensity (Figure 1B), but the two signals were mixed together in the merged image (Figure 1C). After deconvolution and 3D reconstruction, the scatter fluorescence distributed on the inter-bands was eliminated and clearer bands were obtained (Figure 1D-1F). The bands of H3K4me2 were similar to the R-like bands of H4 acetylation on human chromosomes [21]. Using the four FISH probes (TAG, CentC, 45S rDNA, and knob 180-bp, Figure 1G), we were able to identify all chromosomes and construct a karyogram (Figure 1H) for Chang7-2 [22]. Based on the FISH karyogram, we constructed a 3D karyogram of H3K4me2 for Chang7-2 (Figure 1I). The banding pattern was specific for each chromosome pair. This method could also be applied to other modified histones and functional proteins and facilitate analysis of the chromosome structure in plants.

### Histone modification distribution patterns along metaphase chromosomes of different maize lines

Plant genomic alterations often occur during breeding, and in particular, hybridization processes [23]. However, whether or not histone modification patterns are changed is not clear. Based on 3D epikaryotypes, we used six histone modification antibodies (euchromatin mark antibodies: H3K4me2, H3K4me3, H3K9ac, H4K5ac; heterochromatin mark antibodies: H3K9me2, H3K27me2) to compare their immunosignals along metaphase chromosomes of four elite maize inbred lines (B73, Mo17, Chang7-2, Zheng58) and a hybrid line ZD958 (Chang7-2×Zheng58). Some genetic variations among the five maize lines have been reported based on whole-genome re-sequencing [24].

**Figure 2:**
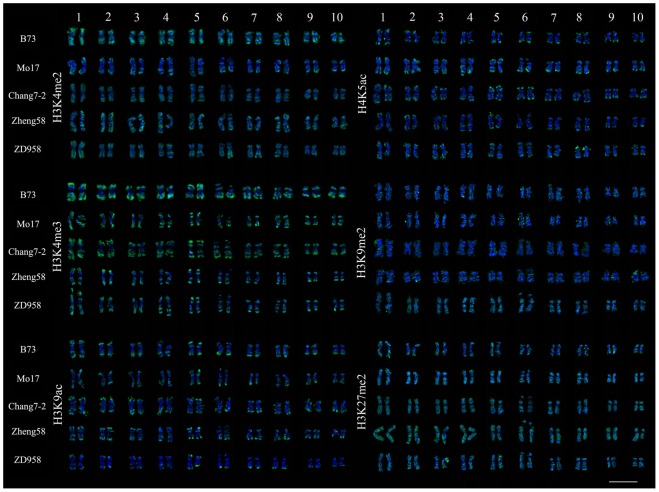
Comparison of the chromosomal distribution of six histone modifications of five maize lines. The 3D immunolabelled (H3K4me2, H3K4me3, H3K9ac, H4K5ac, H3K9me2, and H3K27me2) and DAPI stained chromosomes of four maize inbred lines (B73, Mo17, Chang7-2 and Zheng58) and a hybrid: ZD958 (Chang7-2×Zheng58)) in figure S1 to figure S6 were arranged in the order of the maize chromosome number (1−10). Immunostaining signals in green and DAPI staining signals in blue. Scale bar = 10 µm.

**Figure 3:**
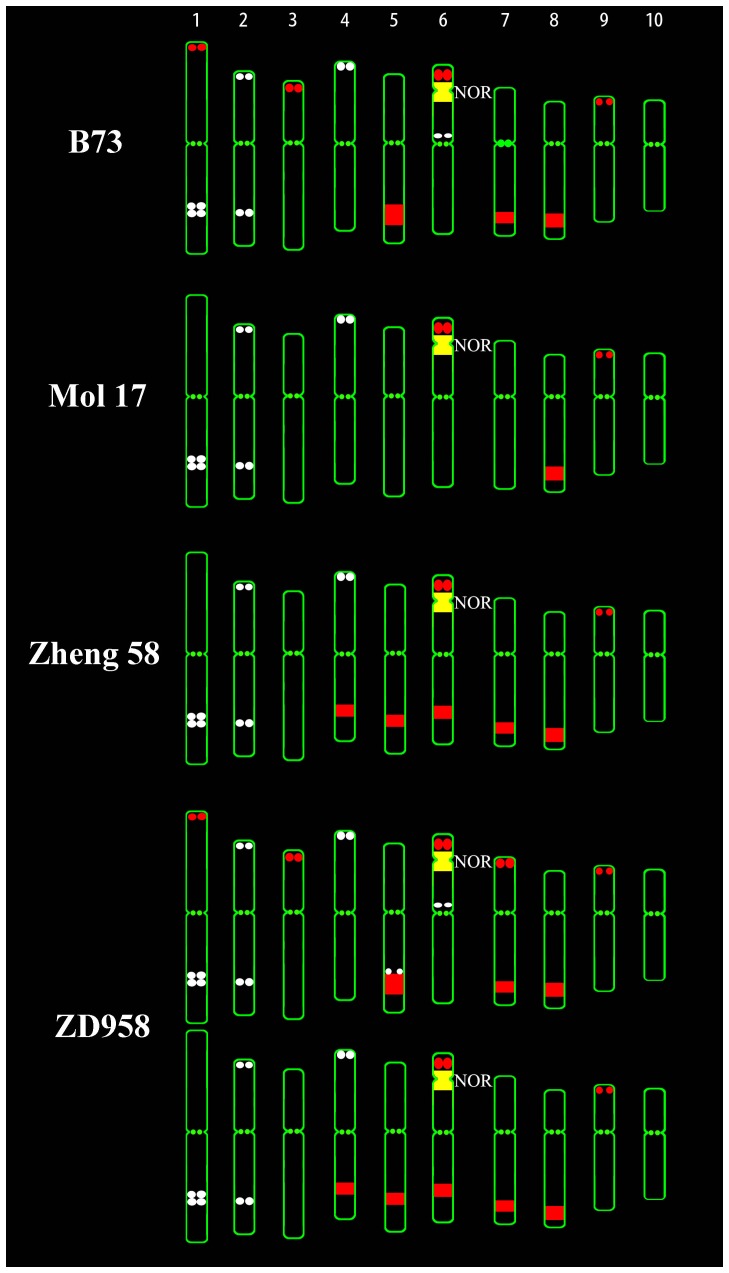
Ideogram of FISH karyotype of four maize lines. The four maize lines include B73, Mo17, Zheng58 and ZD958 (Chang7-2×Zheng58). Assignments of pseudo-colors to each probe: TAG as white, CentC as green, 45S rDNA as yellow and knob 180-bp as red.

**Figure 4:**
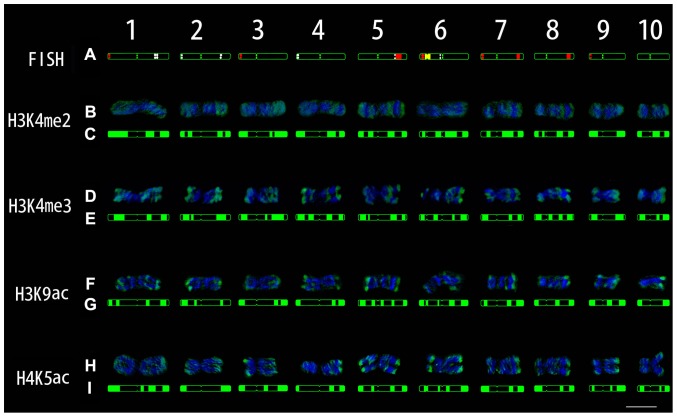
Characterization of chang7-2 metaphase chromosomes by FISH, immunostaining and DAPI signals. (A) FISH karyogram. TAG as white, CentC as green, 45S rDNA as yellow and knob 180-bp as red. (B, D, F and H) Image of immunofluorescence signals (green) and DAPI staining signals (blue) after 3D deconvolution. (C, E. G and I) Immunostaining karyogram. Scale bar = 10 µm.

H3K4me2 is frequently associated with active chromatin [2]. DAPI often strongly stains heterochromatin and A-T rich regions. As expected, the H3K4me2 bands were mainly located at the end of chromosome arms and the regions between heterochromatin. H3K4me2 bands and DAPI bands showed a clear complementary relationship in the inbred line Chang7-2 (Figure 1D and 1F), as most genes are located in the distal region of the chromosome arms in maize and other cereal species [25]. Figure S1 (in supporting information) shows the H3K4me2 staining results of metaphase chromosomes from the other three maize inbred lines (B73, Mo17, and Zheng58) and for the hybrid ZD958. The H3K4me2 distribution patterns of 10 pairs of chromosomes of the four inbred lines and a hybrid line of maize were consistent, as was the immunostaining signal distribution between sister chromatids and homologous chromosomes (Figure 2). This indicates that the H3K4me2 distribution pattern had changed very little at the chromosomal level during breeding and hybridization in maize, although the genome sequence differed between the five maize lines, as indicated by the varying FISH signal distribution of TAG and knob 180-bp probes (Figure 1H, Figure 3, and Figure S1). Taking Chang7-2 as an example, we further compared the relationship between H3K4me2 and the distribution of repetitive sequences. Cytological resolution is insufficient for the examination of histone modifications associated with individual genes or DNA sequences; therefore, we could only examine histone modification levels associated with repetitive sequences. Figure 4 reveals that H3K4me2 was lacking at the TAG, knob 180-bp, 45S rDNA and CentC regions, indicating that H3K4me2 was rarely associated with these repeats.

H3K4me3 and histone acetylation are also often related to active chromatin and gene transcription [2]. Immunostaining by using antibodies against H3K4me3, H3K9ac and H4K5ac showed that the corresponding fluorescence signal patterns on the metaphase chromosomes were similar to those of H3K4me2, with signals gradually weakening from chromosome termini towards the centromeric region (Figures 2, S2, S3 and S4). H3K9ac and H4K5ac signals were additionally found at the nucleolus organizer region (NOR) (Figures S3 and S4, red arrows), which is consistent with the reported result in barley [26]. Signal patterns of these three histone modifications on metaphase chromosomes among the five maize lines were also conservative (Figures 2, S2, S3 and S4). In Figure 4, the signals of H3K4me3 are more enriched than those of H3K4me2, while H3K9ac and H4K5ac signals are weaker than the two methylation signals. Many studies have shown that H3K4me2 is more enriched than H3K4me3 in the animal genome [27]. However, our data in maize are contrary to this observation. TAG, knob 180-bp, and CentC were not mapped within the regions of the three histone modifications, while 45S rDNA was associated with H3K9ac and H4K5ac, possibly indicating transcriptional activity at mitosis.

In some species, H3K9me2 and H3K27me2 are considered to be associated with heterochromatin and gene silencing [2]. Immunostaining experiments using an antibody against H3K9me2 showed that signals were evenly dispersed along the chromosome arm, rather than concentrated on the DAPI deeply stained regions (Figures 2 and S5), which was consistent with the previous result [28]. This is likely to be due to more retrotransposons being present in large genomic plants (>500 Mb), of which the majority are silenced along the chromosomes [28]. H3K27me2 signals were less uniform and mainly located at the chromosome arms, to a lesser degree in some 180-bp knob regions (Figures 2 and S6) and not at centromere regions. The heterochromatin signal patterns on metaphase chromosomes of the five maize lines were also similar (Figures 2, S5 and S6). As the two heterochromatin marks did not form clear bands on metaphase chromosomes, we only constructed the epikaryotypes for the four euchromatin marks H3K4me2, H3K4me3, H3K9ac and H4K5ac in Chang7-2 (Figure 4).

We also wanted to determine whether the expression levels of these histone modifications were the same in the five lines; therefore, we carried out a western blotting experiment. As shown in Figure S7, the expression levels of euchromatin marks H3K4me2, H3K4me3, H3K9ac, H4K5ac and heterochromatin marks H3K9me2, H3K27me2 were almost the same in the different lines, demonstrating that chromosomal distribution and expression levels of these histone modifications remained conserved during selection and hybridization in maize.

### Comparison of distribution of histone modifications along metaphase chromosomes between cultivated maize and its wild relatives

The genus *Zea* includes the cultivated maize and the wild taxa known as teosinte. It has been thought that the cultivated maize (*Z. mays* L. ssp. *mays*) originates from *Z. mays* L. ssp. *Parviglumis* [29]. In this study, we selected two wild relatives (*Z. mays* L. ssp. *parviglumis* and *Z. nicaraguensis*) to investigate whether the chromosomal histone modification patterns have changed during domestication. *Z. mays* L. ssp. *parviglumis* and *Z. mays* L. ssp. *mays* both belong to *Z. mays* [29], while *Z. nicaraguensis* is a different species and shares a distant relationship with *Z. mays* L. ssp. *Mays* [30]. We selected one of the chromatin marks to compare the histone modification distribution patterns between cultivated maize and its wild relatives.

**Figure 5:**
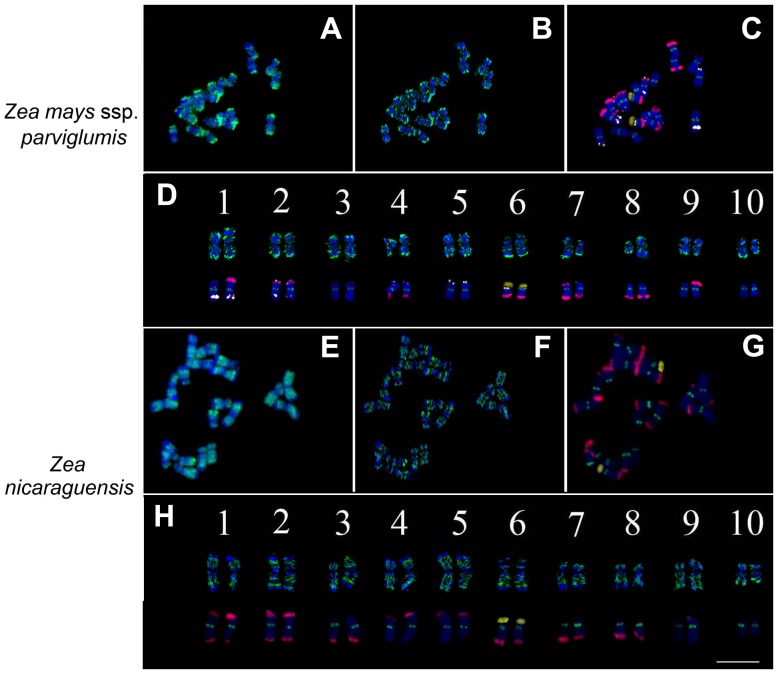
Comparison of H3K4me3 and FISH signal distribution in *Z. mays* ssp. *parviglumis* and *Z. nicaraguensis*. (A, E) 2D image of DAPI staining (blue) signals and H3K4me3 signals (green). (B, F) Image of DAPI staining (blue) signals and H3K4me3 signals (green) after 3D deconvolution. (C, G) Metaphase chromosome identification by combination of the four probes and DAPI staining; Assignments of pseudo-colors to each probe: TAG as white (pseudo-color), CentC as green, 45S rDNA as yellow (pseudo-color) and knob 180-bp as red. (D, H) H3K4me3 and FISH karyotypes in *Z. mays* ssp. *parviglumis* and *Z. nicaraguensis*. Scale bar = 10 µm.

The H3K4me3 signals of *Z. mays* L. ssp. *parviglumis* were mainly located at the chromosome termini, and the distribution pattern was very similar to that of the five cultivated maize lines (Figure 5A, 5B and 5D). However, in *Z. nicaraguensis*, the signals of H3K4me3 were mostly located at the middle of the chromosome arms rather than at the ends, the chromosome termini were strongly DAPI stained, and the H3K4me3 signals were again complementary to the DAPI bands (Figure 5E and 5F and 5H). Thus, H3K4me3 represents a euchromatin marker in *Z. nicaraguensis*, but the signal distribution pattern differs from that of *Z. mays* L. ssp. *parviglumis* and the five cultivated maize lines (Figure 2). FISH signals of 45S rDNA, CentC, TAG and knob 180-bp could be detected in *Z. mays* L. ssp. *parviglumis* (Figure 5C), but no signal for TAG was detectable in *Z. nicaraguensis* (Figure 5G), confirming that cultivated maize is closer related to *Z. mays* L. ssp. *parviglumis* than to *Z. nicaraguensis*. The H3K4me3 signals were mainly located at the end of chromosomes and the knob 180-bp signals were interstitial in *Z. mays* L. ssp. *parviglumis* (Figure 5D) and the maize inbred line Zheng58 (Figures 1, 2 and 3), while the knob but not the H3K4me3 signals were at the end of chromosomes in *Z. nicaraguensis* (Figure 5H).

### Comparison of histone modification patterns between metaphase chromosomes and interphase nuclei

The chromatin structure gradually becomes more compact as cells enter mitosis and histone post-translational modifications play an important role in establishing higher order chromatin folding. Previous studies have shown that some of the histone modifications of the interphase genome are retained in human metaphase chromosomes [3]. Recently, genome-wide landscapes of histone modifications in maize interphase nuclei have been defined using ChIP-seq [31]. The results could be aligned with histone modification patterns across metaphase chromosomes for the comparison of interphase and metaphase histone modification states.

**Figure 6:**
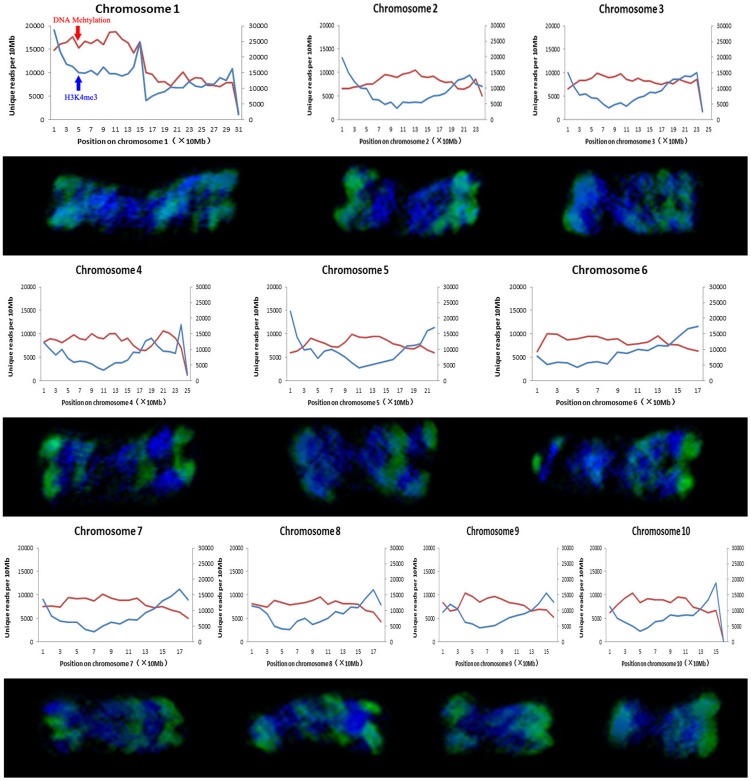
Comparison of H3K4me3 distribution of across interphase nuclei and metaphase chromosomes in B73. Immunolabelled metaphase chromosomes (DAPI staining signals in blue and immunosignals in green) by H3K4me3 are aligned with the distribution of H3K4me3 and DNA methylation in the equivalent interphase nuclei assembled from ChIP-seq data [31]. Each chromosome was divided into 10 Mb intervals. The left vertical axis indicates the number of unique reads per 10 Mb of H3K4me3 (blue curve), and the right vertical axis indicates the number of unique reads per 10 Mb of DNA methylation (red curve).

**Figure 7:**
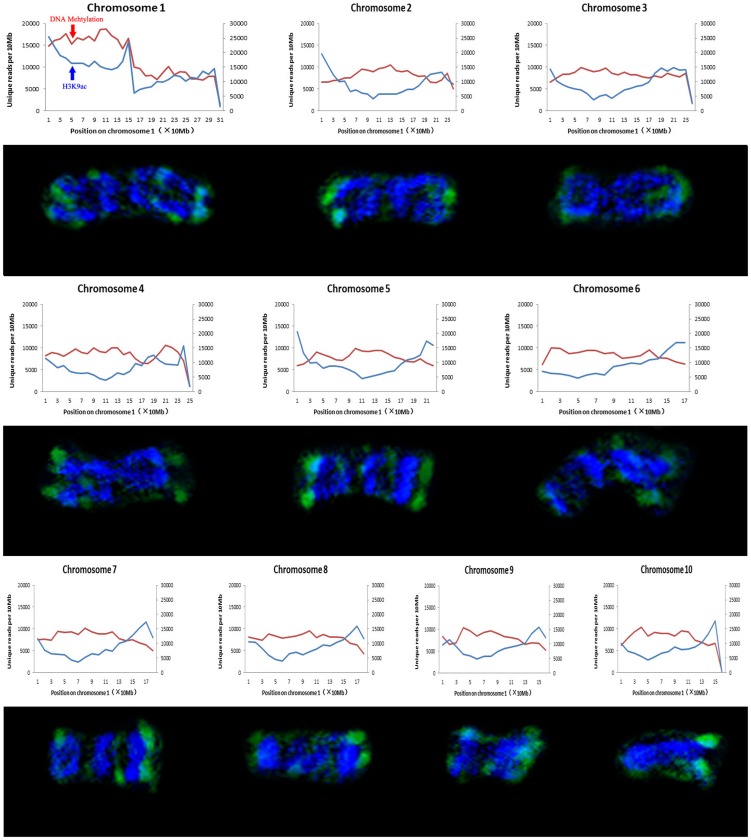
Comparison of the distribution of H3K9ac across interphase nuclei and metaphase chromosomes in B73. Immunolabelled metaphase chromosomes (DAPI staining signals in blue and immunosignals in green) by H3K9ac are aligned with the distribution of H3K9ac and DNA methylation in the equivalent interphase nuclei assembled from ChIP-seq data [31]. Each chromosome was divided into 10 Mb intervals. The left vertical axis indicates the number of unique reads per 10 Mb of H3K9ac (blue curve), and the right vertical axis indicates the number of unique reads per 10 Mb of DNA methylation (red curve).

The maize genome (∼3,000 Mbp) consists of 80% or more repetitive sequences, meaning that it is difficult to accurately map all ChIP-seq reads to the maize genome [16]. Therefore, we only selected the reads that mapped to unique positions and compared the histone modification patterns of non-repetitive sequence at interphase and metaphase. As shown in Figures 6 and 7, major immunostaining bands for H3K4me3 and H3K9ac on metaphase chromosomes correspond to the peaks defined at interphase by ChIP-seq at 10-Mb resolution, especially at the chromosome ends. However, as cells enter mitosis, some epigenetic signals observed in interphase disappeared on metaphase chromosomes. For example, the peaks of H3K4me3 and H3K9ac signals appeared to be located close to the centromeres on chromosome 1 at interphase, but were absent from chromosome 1 at metaphase (Figures 6 and 7). H3K4me3 and H3K9ac signals were mainly concentrated on the chromosome ends that are gene-rich regions (Figures 6 and 7). Therefore, some signals may be preferentially removed from certain gene-poor regions during transition from interphase to metaphase. ChIP-seq results revealed no detectable differences with regard to the distribution of H3K4me3 and H3K9ac at interphase, but H3K4me3 seemed to be more abundant than H3K9ac on metaphase chromosomes. This suggested that histone-3 deacetylation at lysine-9 occurred towards mitosis, which is consistent with the results of previous studies [32]. DNA methylation is a heterochromatin mark [2]. Our comparative results also showed that the DNA methylation distribution was complementary to H3K4me3 and H3K9ac signals at both metaphase and interphase.

**Figure 8:**
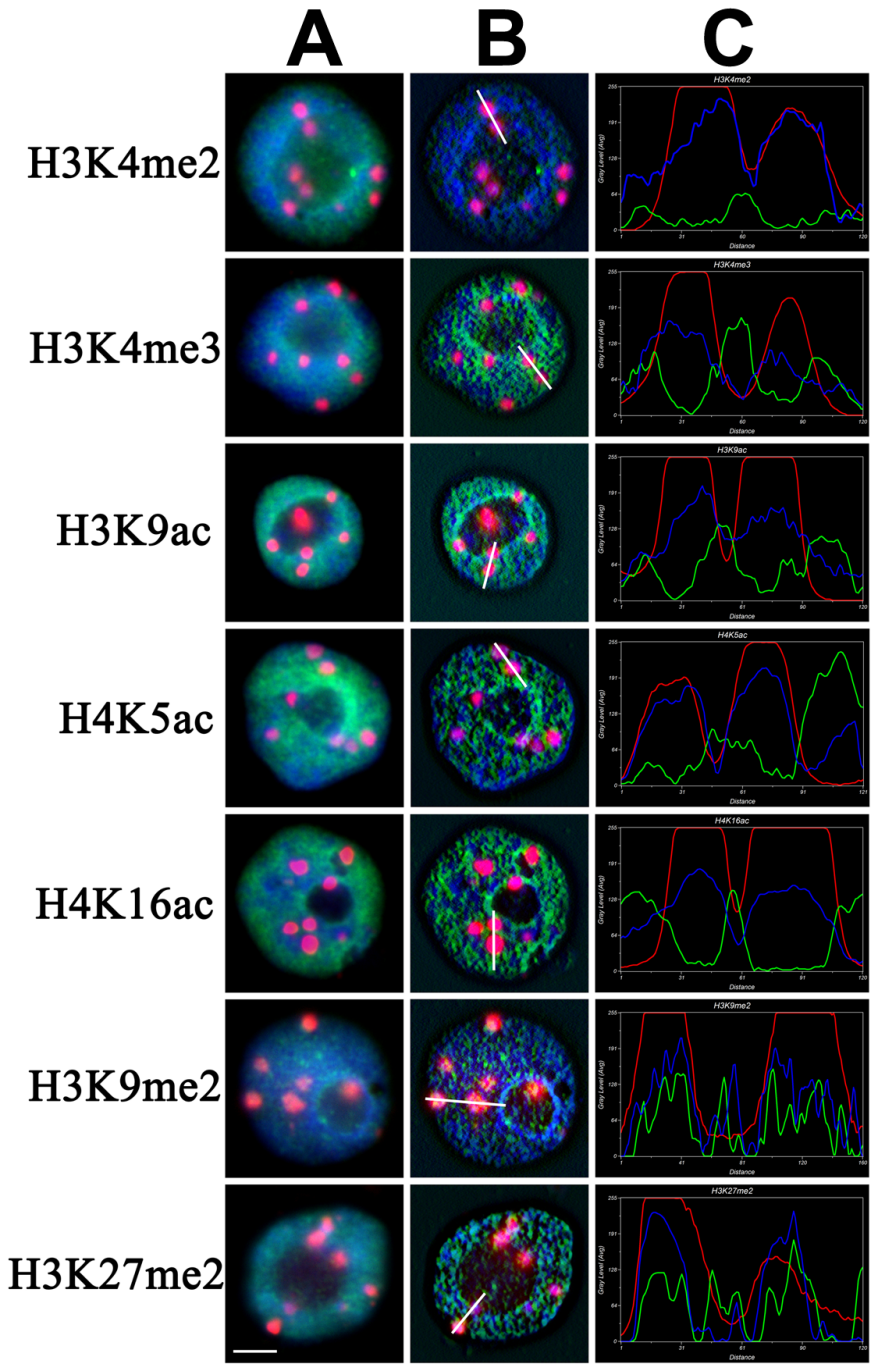
Histone modifications at 180-bp knobs in chang7-2 root nuclei. (A) 2D image. DAPI staining signals in blue, immunosignals in green and knob 180-bp signals in red. (B) Image after 3D deconvolution. (C) The intensity signals along the white line. The horizontal axis indicates the location of the selected chromatin, and the vertical axis indicates the grey level. Scale bar = 5 µm.

In order to compare the histone modifications at repetitive sequences during interphase and metaphase, heterochromatic knobs, which consist of tandem repeats [22], were examined for histone modifications of knob 180-bp repeats at interphase nuclei (Figure 8). Compared with the original image (Figure 8A), the three-dimensionally reconstructed image after deconvolution clearly showed that the euchromatin marks (H3K4me2, H3K4me3, H3K9ac and H4K5ac) did not stain the knob 180-bp regions, while heterochromatin marks (H3K9me2 and H3K27me2) were present in the 180-bp knob regions (Figure 8B). The fluorescence curve could directly reflect the relationship between DAPI, histone modification and knob 180-bp signals (Figure 8C). The fluorescence curves of the euchromatin mark signals (H3K4me2, H3K4me3, H3K9ac and H4K5ac) were complementary to those of DAPI and 180-bp knob signals. The fluorescence curves of the heterochromatin mark signals (H3K9me2 and H3K27me2) were consistent with those of DAPI and 180-bp knob signals. The results in interphase nuclei were consistent with those observed on metaphase chromosomes (Figure 4), suggesting that the epigenetic memories associated with repetitive sequences were retained as the cell progressed into metaphase from interphase.

## Discussion

### 3D epikaryotyping for the analysis of modified histones or methylated DNA at the single chromosome level

Chromosome banding karyotypes using various staining techniques reveal characteristic patterns of transverse bands along chromosomes, allowing for the identification of chromosomes and the analysis of chromosome organization and evolutionary relationships [20]. Immunolabelling of human metaphase chromosomes with modified histones can also generate unique banding patterns [3]. However, it is difficult to produce identifiable bands in plant chromosomes stained by various dyes due to the coarseness in the pattern of the bands. The development of computer image processing and analysis techniques, especially the technique of 3D reconstruction and deconvolution, could improve banding resolution and offer opportunities to obtain clear bands [33]. In maize meiotic pachytene chromosomes, 3D imaging data provided a complete view of various histone methylation distributions [12]. Metaphase chromosomes are more condensed than pachytene chromosomes and the global trends of histone modifications are easier to detect; thus, these features could facilitate the comparison of histone modification patterns between different lines or species [22]. The 3D epikaryotyping technique provides valuable chromosome-scale view of modified histones or methylated DNA at the single chromosome level, and provides a link between chromosomes, epigenomes and DNA sequences through combination with ChIP-seq data, and can also solve the problems encountered by the ChIP-based approach.

Our results in figure 2 revealed that the distribution patterns of the histone modifications for euchromatin marks across metaphase chromosomes were very similar to those on pachytene chromosomes in maize, while the distribution patterns of H3K9me2 and H3K27me2 showed differences between metaphase and pachytene chromosomes [12]. H3K9me2 signals were distributed across all chromosomes, while H3K27me2 signals were mainly concentrated at the chromosome arms with a reduced level at knob regions. H3K27me2 may not be a typical heterochromatin mark in maize. These results are consistent with a previous study on maize metaphase chromosomes [10], whereas in maize meiosis pachytene, H3K9me2 is associated with euchromatin and H3K27me2 marks classical heterochromatin [12]. We do not know the reason for these differences, but it is possible that some histone modifications are reprogrammed at gametogenesis and fertilization [34]. Further investigations are needed to understand the differential histone modification distribution patterns between pachytene meiosis chromosomes and metaphase chromosomes.

### Euchromatic histone modifications may be maintained throughout the cell cycle

Histone modifications are known to carry important epigenetic information and play an important role in the control of nucleosome folding into a higher order structure and the regulation of gene expression [1]. Our results showed that some histone modification bands on maize metaphase chromosomes correspond to defined peaks for interphase chromatin as revealed by ChIP-seq and immunostaining signals in nuclei, although histone modifications may vary during the progression from interphase to metaphase (Figures 6 and 7). These results are consistent with previous reports in humans [3]. H3K4me3 and H3K9ac are thought to be associated with active chromatin and gene transcription. We found that some signals of H3K4me3 and especially H3K9ac may be preferentially removed from certain gene-poor regions at metaphase. Previous studies have shown that the overall level of histone acetylation gradually declines in the process of mitosis and the removal of the acetyl group is necessary for chromatin condensation [35]. Due to low transcriptional activity at metaphase, some euchromatic histone modifications may be removed from the chromatin and return to the origin level at telophase for gene reactivation in the next cycle. Unlike strict semi-conservative DNA replication, epigenetic modifications are dynamically re-established during each mitotic cycle [36].

Histone modifications may function as molecular marks for the recruitment of activators and repressors to specific locations of the genome to maintain gene expression patterns, and the maintenance of histone modifications could be a way for the cell to propagate different states in different chromatin regions during cell division [37]. During X-chromosome inactivation and parental imprinting, each allele of a pair is differently regulated in the same nucleus. It has been demonstrated that the genes themselves are marked by different DNA methylation and histone modifications, implying that these epigenetic modifications have an effect on gene expression [38].

### Chromosomal histone modification patterns are retained during maize domestication

About ten thousand years ago, people began to domesticate crops, and crossed them with other domesticated lines or with wild relatives to produce varieties with desirable properties. During domestication and breeding, selected new phenotypes were considered to be due to genetic variation among populations. However, recent advances have drawn attention to epigenetic variation, which can also lead to phenotypic diversity and can be heritable across generations [39]. It was reported that substantial DNA methylation variation exists between different *A. thaliana* accessions [40], whereas H3K4me2 and H3K27me3 distribution patterns are highly conserved and remain largely unchanged in their F1 hybrids [41]. DNA methylation variation was also found in two rice subspecies (*Oryza sativa japonica* and *O. sativa indica*) and their reciprocal hybrids, but H3K4me3 and H3K27me3 levels were not found to be drastically changed [42]. Histone modification patterns seem to be conserved compared with DNA methylation, and may be inherited through generations to influence the phenotypes. In maize, there were no obvious differences in the global epigenetic modification distribution patterns in different genotypes and organs, but there were more substantial differences in histone modification between organs than between genotypes and their hybrids; however, DNA methylations were similar between organs but distinct between different genotypes and their hybrids [43], [44].

Our comparative analysis of epikaryotypes revealed that the histone modification distribution patterns were very similar for different maize inbred lines, a hybrid line and their progenitor *Z. mays* L. ssp. *parviglumis*, but differed from that of *Z. nicaraguensis*. *Z. nicaraguensis*, another wild teosinte, more distantly related to cultivated maize, has larger chromosomes with terminal knobs, while cultivated maize and *Z. mays* L. ssp. *parviglumis* mostly have subterminal knobs [30]. Similar chromosomal distribution patterns for cultivated maize and *Z. mays* L. ssp. *parviglumis* demonstrated that the chromosomal distribution patterns of histone modifications have not changed much during domestication (Figures 2 and 5). Although the genome sequences differ slightly between the five maize lines and *Z. mays* L. ssp. *Parviglumis* according to our FISH data and the whole-genome re-sequencing results [24], chromosome morphology and DAPI staining patterns are very similar, revealing no detectable change during maize domestication. Differences in chromosome structure may be one of the reasons for deviating histone modification patterns between *Z. nicaraguensis* and cultivated maize. Our results demonstrated that histone modification distribution patterns are conserved at the chromosomal level during maize evolution, which is concordant with reports for maize, rice and *A. thaliana* at the molecular level [41]-[44], and confirmed the origin of cultivated maize to be *Z. mays* L. ssp. *parviglumis* [29].

## Supporting Information

Figure S1 **Chromosomal distribution of H3K4me2 between four maize inbred lines and a hybrid.** (a1–a5) 2D image of DAPI staining signals (blue). (b1–b5) 2D image of immunofluorescence signals (green). (c1–c5) Mergers of (a1–a5) and (b1–b5), respectively. (d1–d5) Image of DAPI staining signals after 3D deconvolution (blue). (e1–e5) Image of immunofluorescence signals after 3D deconvolution (green). (f1–f5) Merges of (d1–d5) and (e1–e5) respectively. (g1–g5) FISH with four probes performed after H3K4me3 immunolabelling. Assignments of pseudo-colors to each probe: TAG as white, CentC as green, 45s rDNA as yellow and knob 180-bp as red. Scale bar = 10 µm.
(JPG)

Figure S2 **Chromosomal distribution of H3K4me3 between four maize inbred lines and a hybrid.** (a1–a5) 2D image of DAPI staining signals (blue). (b1–b5) 2D image of immunofluorescence signals (green). (c1–c5) Mergers of (a1–a5) and (b1–b5), respectively. (d1–d5) Image of DAPI staining signals after 3D deconvolution (blue). (e1–e5) Image of immunofluorescence signals after 3D deconvolution (green). (f1–f5) Mergers of (d1–d5) and (e1–e5), respectively. (g1–g5) FISH with four probes performed after H3K4me3 immunolabelling. Assignments of pseudo-colors to each probe: TAG as white, CentC as green, 45s rDNA as yellow and knob 180-bp as red. Scale bar = 10 µm.
(JPG)

Figure S3 **Chromosomal distribution of H3K9ac between four maize inbred lines and a hybrid.** (a1–a5) 2D image of DAPI staining signals (blue). (b1–b5) 2D image of immunofluorescence signals (green). (c1–c5) Mergers of (a1–a5) and (b1–b5), respectively. NORs are indicated by red arrows. (d1–d5) Image of DAPI staining signals after 3D deconvolution (blue). (e1–e5) Image of immunofluorescence signals after 3D deconvolution (green). (f1–f5) Merge of (d1–d5) and (e1–e5) respectively. NORs are indicated by red arrows. (g1–g5) FISH with four probes performed after H3K4me3 immunolabelling. Assignments of pseudo-colors to each probe: TAG as white, CentC as green, 45s rDNA as yellow and knob 180-bp as red. Scale bar = 10 µm.
(JPG)

Figure S4 **Chromosomal distribution of H4K5ac between four maize inbred lines and a hybrid.** (a1–a5) 2D image of DAPI staining signals (blue). (b1–b5) 2D image of immunofluorescence signals (green). (c1–c5) Mergers of (a1–a5) and (b1–b5), respectively. NORs are indicated by red arrows. (d1–d5) Image of DAPI staining signals after 3D deconvolution (blue). (e1–e5) Image of immunofluorescence signals after 3D deconvolution (green). (f1–f5) Mergers of (d1–d5) and (e1–e5), respectively. NORs are indicated by red arrows. (g1–g5) FISH with four probes performed after H3K4me3 immunolabelling. Assignments of pseudo-colors to each probe: TAG as white, CentC as green, 45s rDNA as yellow and knob 180-bp as red. Scale bar = 10 µm.
(JPG)

Figure S5 **Chromosomal distribution of H3K9me2 between four maize inbred lines and a hybrid.** (a1–a5) 2D image of DAPI staining signals (blue). (b1–b5) 2D image of immunofluorescence signals (green). (c1–c5) Mergers of (a1–a5) and (b1–b5), respectively. (d1–d5) Image of DAPI staining signals after 3D deconvolution (blue). (e1–e5) Image of immunofluorescence signals after 3D deconvolution (green). (f1–f5) Mergers of (d1–d5) and (e1–e5), respectively. (g1–g5) FISH with four probes performed after H3K4me3 immunolabelling. Assignments of pseudo-colors to each probe: TAG as white, CentC as green, 45s rDNA as yellow and knob 180-bp as red. Scale bar = 10 µm.
(JPG)

Figure S6 **Chromosomal distribution of H3K27me2 between four maize inbred lines and a hybrid.** (a1–a5) 2D image of DAPI staining signals (blue). (b1–b5) 2D image of immunofluorescence signals (green). (c1–c5) Mergers of (a1–a5) and (b1–b5), respectively. (d1–d5) 3 Image of DAPI staining signals after 3D deconvolution (blue). (e1–e5) Image of immunofluorescence signals after 3D deconvolution (green). (f1–f5) Mergers of (d1–d5) and (e1–e5), respectively. (g1–g5) FISH with four probes performed after H3K4me3 immunolabelling. Assignments of pseudo-colors to each probe: TAG as white, CentC as green, 45s rDNA as yellow and knob 180-bp as red. Scale bar = 10 µm.
(JPG)

Figure S7 **Comparison of expression levels of histone modification between four maize inbred lines and a hybrid.** Histone modifications include H3K4me2, H3K4me3, H3K9ac, H4K5ac, H3K9me2 and H3K27me2. Histone H3 was applied as an equal loading control. 1: B73, 2: Mo17, 3: Chang7-2, 4: Zheng58, 5: ZD958.
(JPG)
