# Supplementary figures and images for: Comparative Analysis of Genome-Wide Chromosomal Histone Modification Patterns in Maize Cultivars and Their Wild Relatives
Source: PLoS One. 2014 May 12;9(5):e97364. doi: 10.1371/journal.pone.0097364 (PMC4018347; doi:10.1371/journal.pone.0097364)

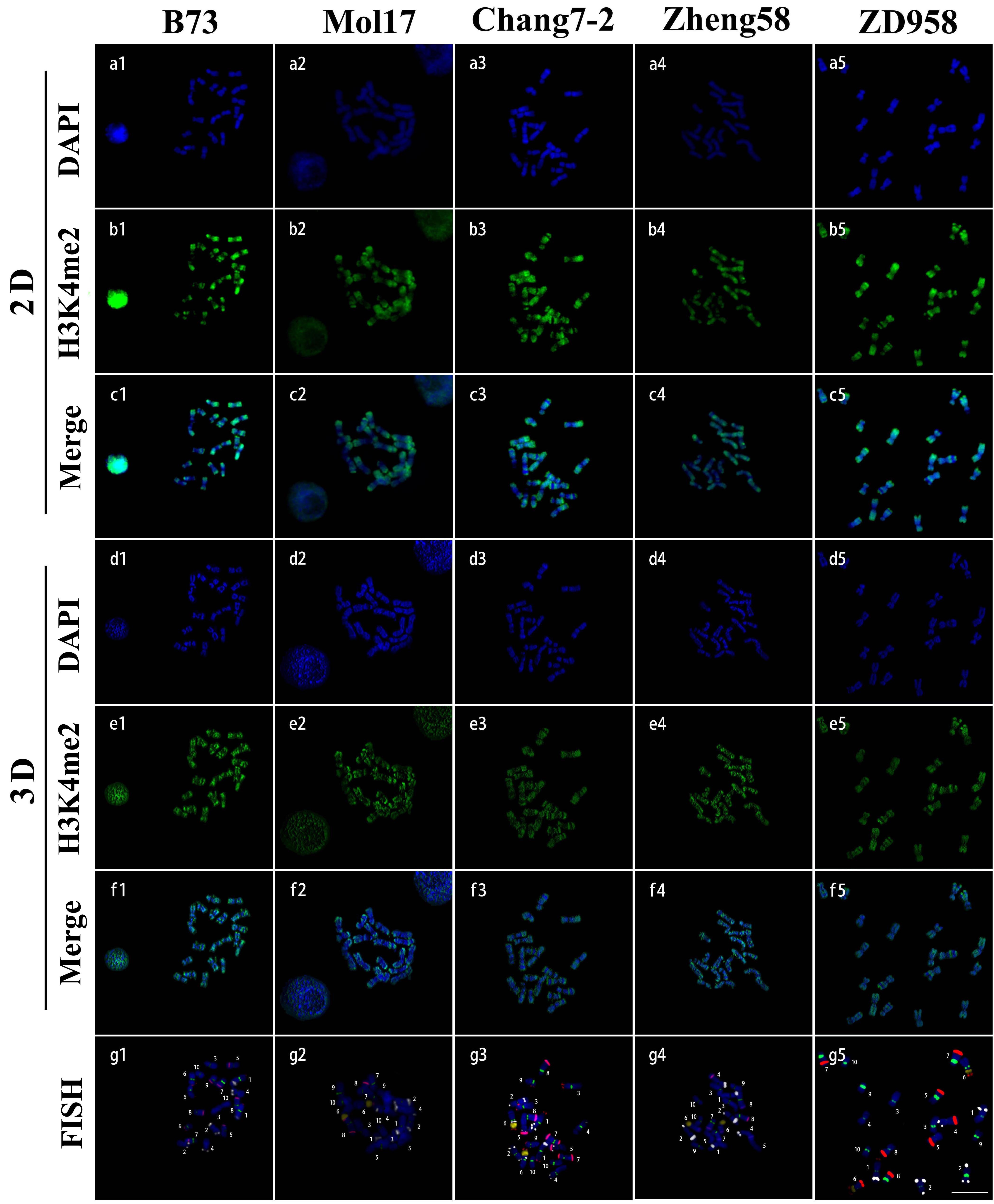

Supplement: Figure S1 — Chromosomal distribution of H3K4me2 between four maize inbred lines and a hybrid. (a1–a5) 2D image of DAPI staining signals (blue). (b1–b5) 2D image of immunofluorescence signals (green). (c1–c5) Mergers of (a1–a5) and (b1–b5), respectively. (d1–d5) Image of DAPI staining signals after 3D deconvolution (blue). (e1–e5) Image of immunofluorescence signals after 3D deconvolution (green). (f1–f5) Merges of (d1–d5) and (e1–e5) respectively. (g1–g5) FISH with four probes performed after H3K4me3 immunolabelling. Assignments of pseudo-colors to each probe: TAG as white, CentC as green, 45s rDNA as yellow and knob 180-bp as red. Scale bar = 10 µm. (JPG) [file pone.0097364.s001.jpg]

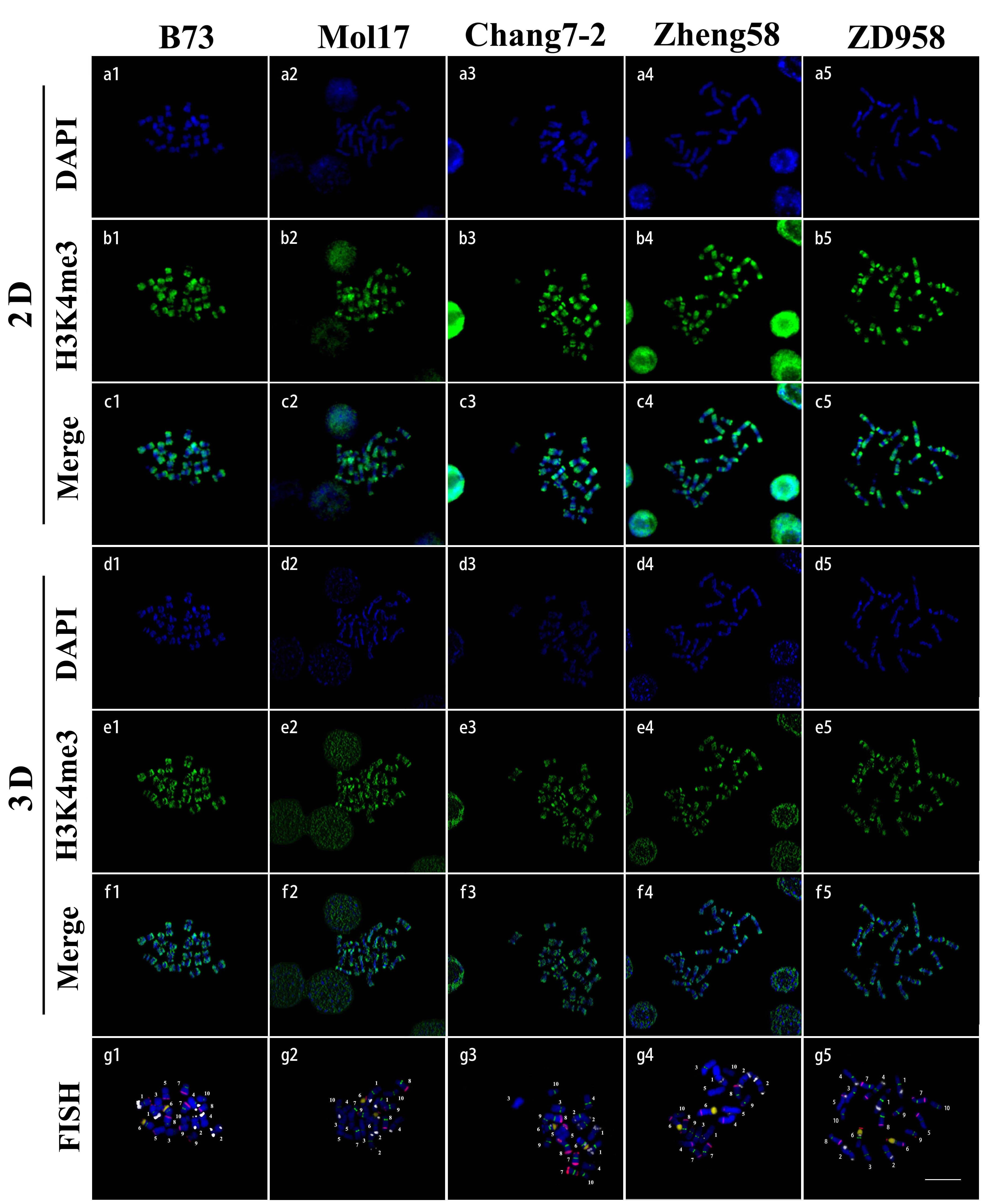

Supplement: Figure S2 — Chromosomal distribution of H3K4me3 between four maize inbred lines and a hybrid. (a1–a5) 2D image of DAPI staining signals (blue). (b1–b5) 2D image of immunofluorescence signals (green). (c1–c5) Mergers of (a1–a5) and (b1–b5), respectively. (d1–d5) Image of DAPI staining signals after 3D deconvolution (blue). (e1–e5) Image of immunofluorescence signals after 3D deconvolution (green). (f1–f5) Mergers of (d1–d5) and (e1–e5), respectively. (g1–g5) FISH with four probes performed after H3K4me3 immunolabelling. Assignments of pseudo-colors to each probe: TAG as white, CentC as green, 45s rDNA as yellow and knob 180-bp as red. Scale bar = 10 µm. (JPG) [file pone.0097364.s002.jpg]

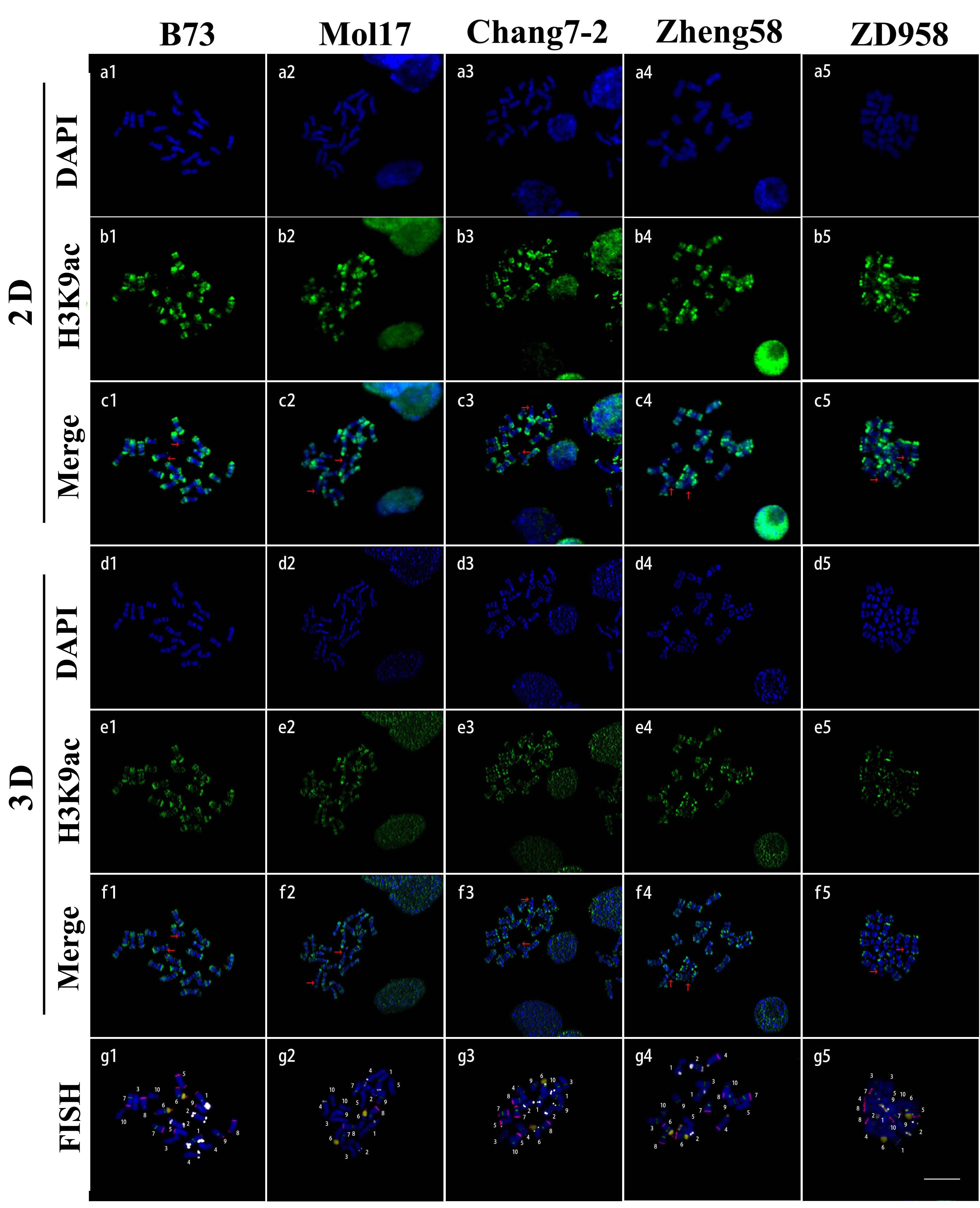

Supplement: Figure S3 — Chromosomal distribution of H3K9ac between four maize inbred lines and a hybrid. (a1–a5) 2D image of DAPI staining signals (blue). (b1–b5) 2D image of immunofluorescence signals (green). (c1–c5) Mergers of (a1–a5) and (b1–b5), respectively. NORs are indicated by red arrows. (d1–d5) Image of DAPI staining signals after 3D deconvolution (blue). (e1–e5) Image of immunofluorescence signals after 3D deconvolution (green). (f1–f5) Merge of (d1–d5) and (e1–e5) respectively. NORs are indicated by red arrows. (g1–g5) FISH with four probes performed after H3K4me3 immunolabelling. Assignments of pseudo-colors to each probe: TAG as white, CentC as green, 45s rDNA as yellow and knob 180-bp as red. Scale bar = 10 µm. (JPG) [file pone.0097364.s003.jpg]

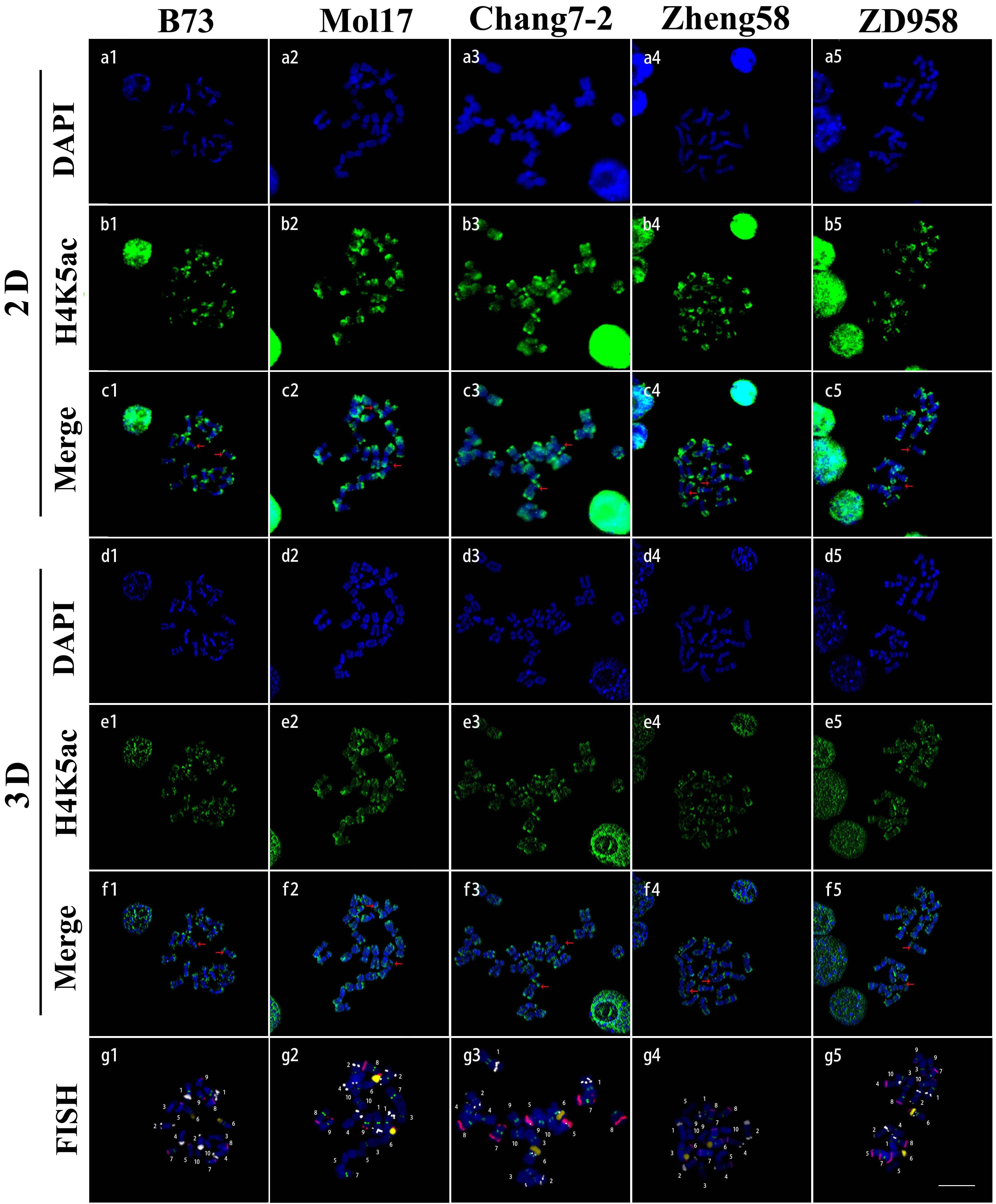

Supplement: Figure S4 — Chromosomal distribution of H4K5ac between four maize inbred lines and a hybrid. (a1–a5) 2D image of DAPI staining signals (blue). (b1–b5) 2D image of immunofluorescence signals (green). (c1–c5) Mergers of (a1–a5) and (b1–b5), respectively. NORs are indicated by red arrows. (d1–d5) Image of DAPI staining signals after 3D deconvolution (blue). (e1–e5) Image of immunofluorescence signals after 3D deconvolution (green). (f1–f5) Mergers of (d1–d5) and (e1–e5), respectively. NORs are indicated by red arrows. (g1–g5) FISH with four probes performed after H3K4me3 immunolabelling. Assignments of pseudo-colors to each probe: TAG as white, CentC as green, 45s rDNA as yellow and knob 180-bp as red. Scale bar = 10 µm. (JPG) [file pone.0097364.s004.jpg]

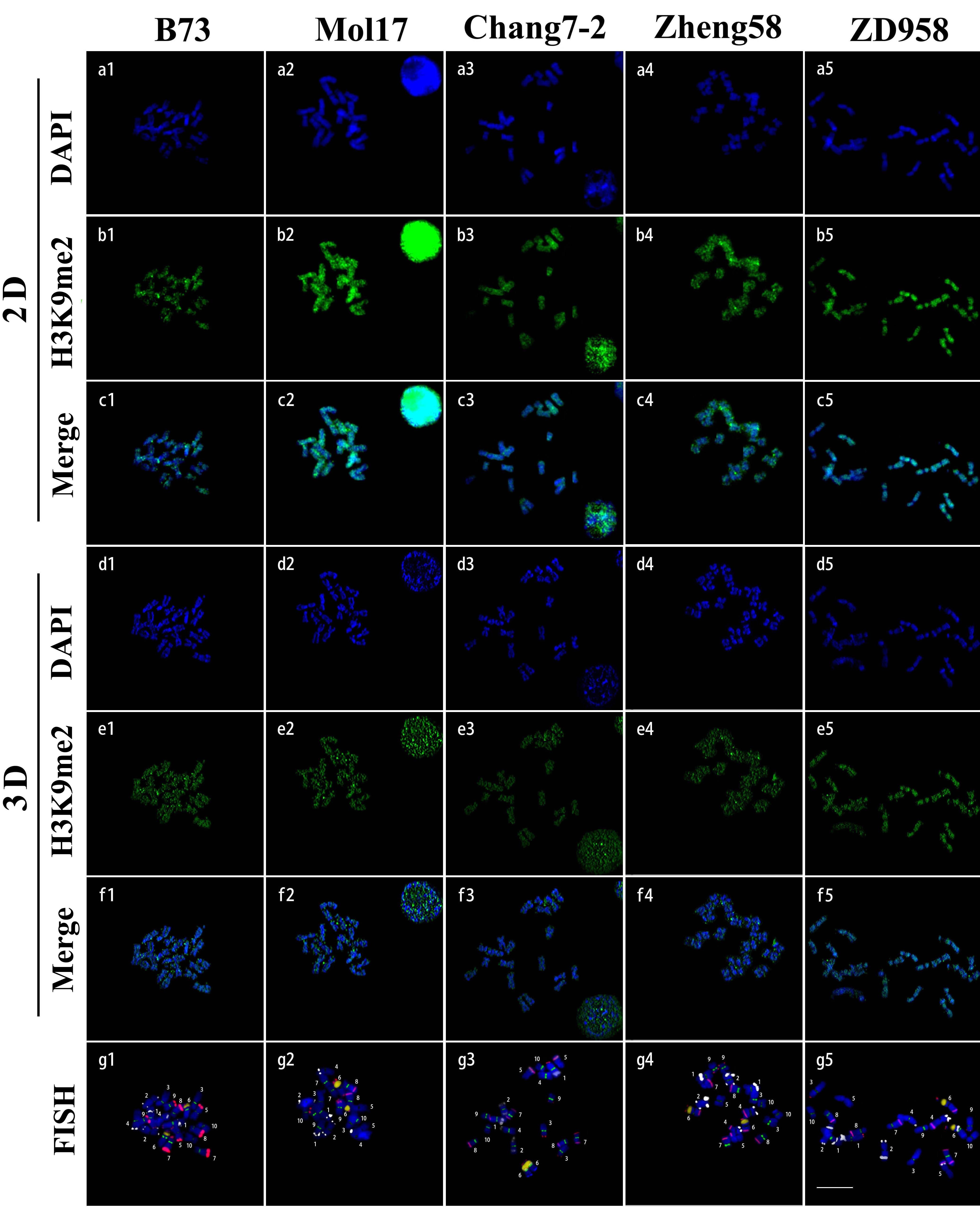

Supplement: Figure S5 — Chromosomal distribution of H3K9me2 between four maize inbred lines and a hybrid. (a1–a5) 2D image of DAPI staining signals (blue). (b1–b5) 2D image of immunofluorescence signals (green). (c1–c5) Mergers of (a1–a5) and (b1–b5), respectively. (d1–d5) Image of DAPI staining signals after 3D deconvolution (blue). (e1–e5) Image of immunofluorescence signals after 3D deconvolution (green). (f1–f5) Mergers of (d1–d5) and (e1–e5), respectively. (g1–g5) FISH with four probes performed after H3K4me3 immunolabelling. Assignments of pseudo-colors to each probe: TAG as white, CentC as green, 45s rDNA as yellow and knob 180-bp as red. Scale bar = 10 µm. (JPG) [file pone.0097364.s005.jpg]

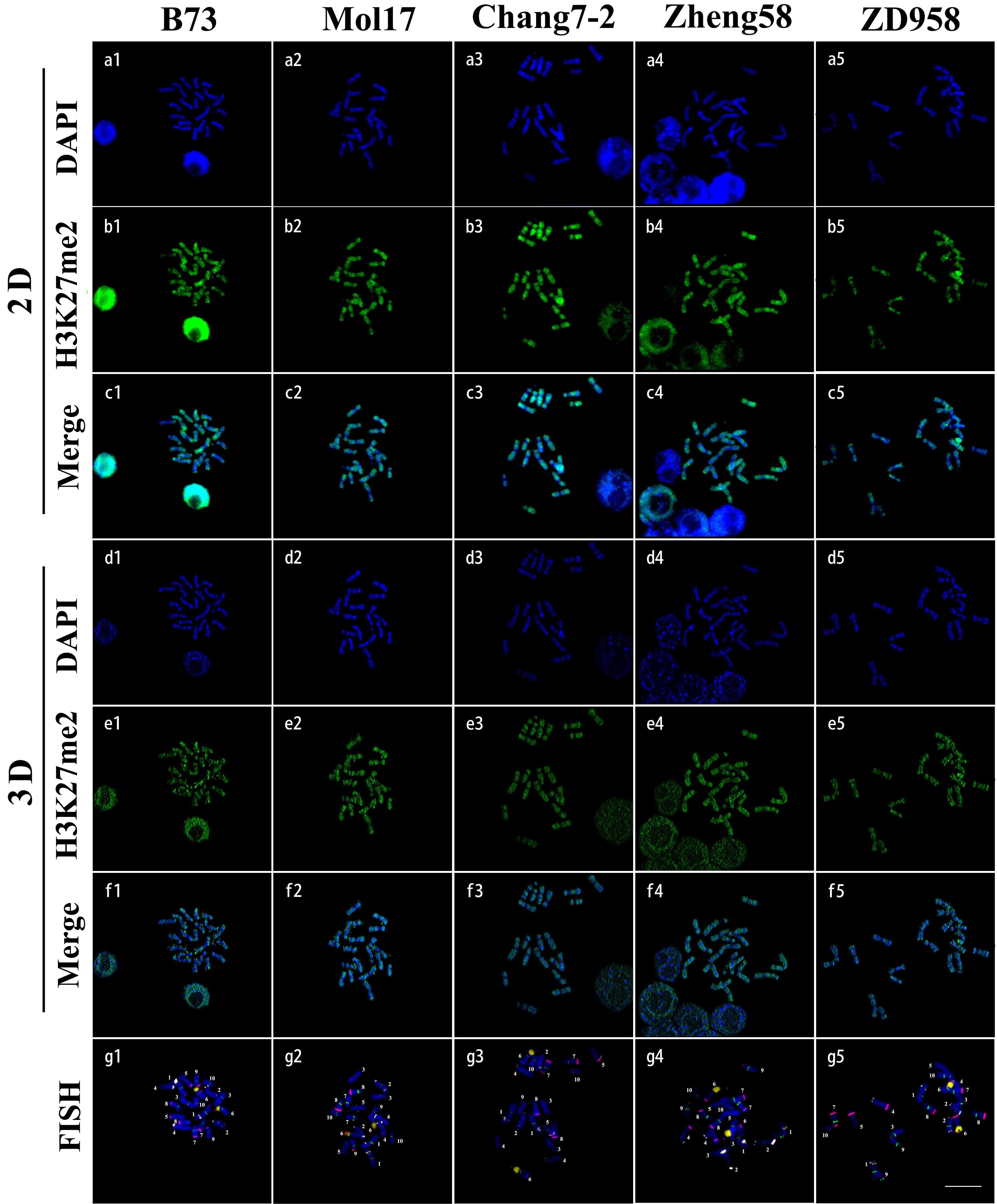

Supplement: Figure S6 — Chromosomal distribution of H3K27me2 between four maize inbred lines and a hybrid. (a1–a5) 2D image of DAPI staining signals (blue). (b1–b5) 2D image of immunofluorescence signals (green). (c1–c5) Mergers of (a1–a5) and (b1–b5), respectively. (d1–d5) 3 Image of DAPI staining signals after 3D deconvolution (blue). (e1–e5) Image of immunofluorescence signals after 3D deconvolution (green). (f1–f5) Mergers of (d1–d5) and (e1–e5), respectively. (g1–g5) FISH with four probes performed after H3K4me3 immunolabelling. Assignments of pseudo-colors to each probe: TAG as white, CentC as green, 45s rDNA as yellow and knob 180-bp as red. Scale bar = 10 µm. (JPG) [file pone.0097364.s006.jpg]

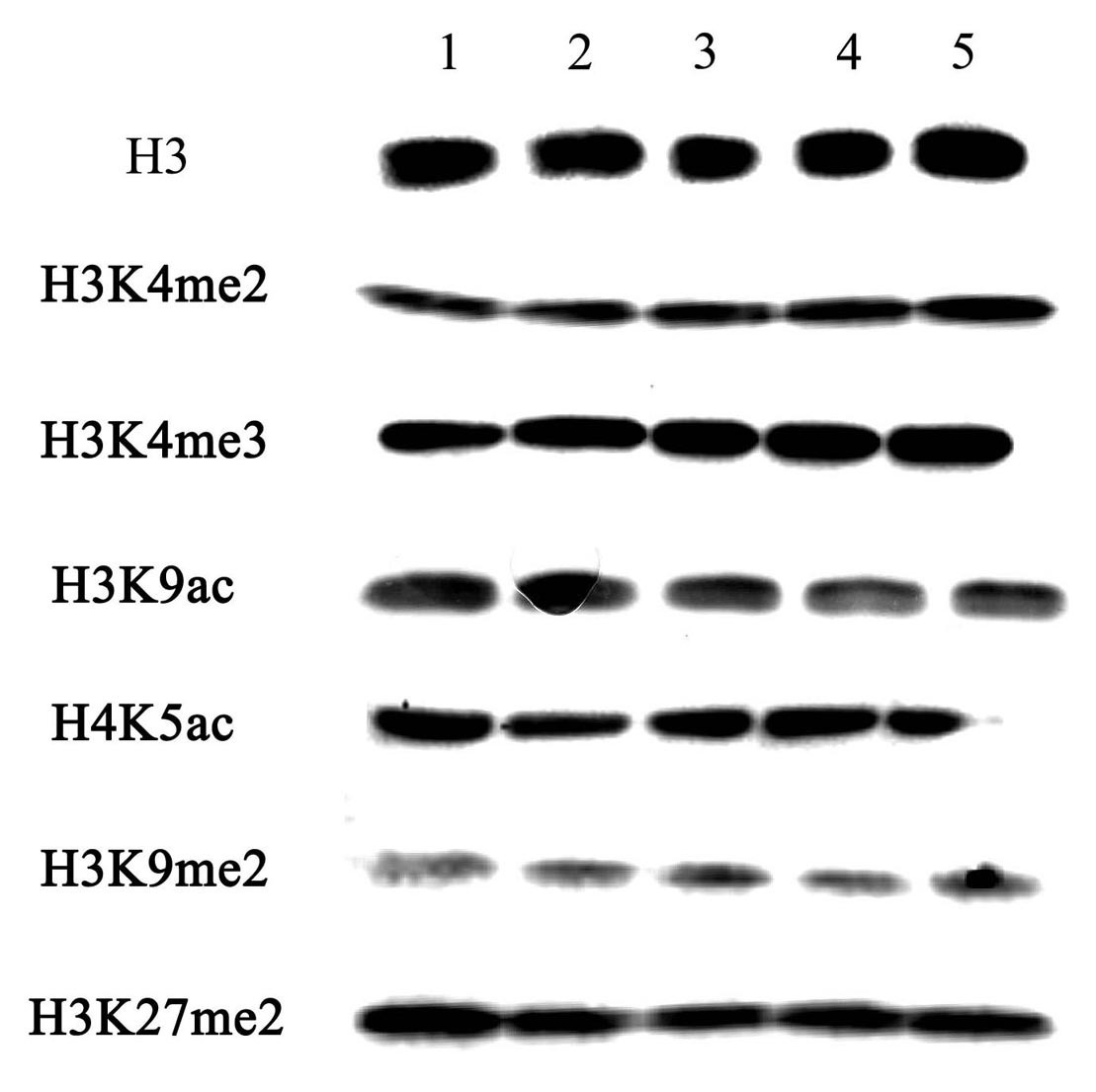

Supplement: Figure S7 — Comparison of expression levels of histone modification between four maize inbred lines and a hybrid. Histone modifications include H3K4me2, H3K4me3, H3K9ac, H4K5ac, H3K9me2 and H3K27me2. Histone H3 was applied as an equal loading control. 1: B73, 2: Mo17, 3: Chang7-2, 4: Zheng58, 5: ZD958. (JPG) [file pone.0097364.s007.jpg]
